# Blending Samples to Increase Accuracy and Precision of ^1^H NMR Urine Metabolomics

**DOI:** 10.1021/acs.analchem.4c01532

**Published:** 2024-07-31

**Authors:** Anders Bay Nord, Helen Lindqvist, Millie Rådjursöga, Anna Winkvist, B. Göran Karlsson, Daniel Malmodin

**Affiliations:** † Swedish NMR Centre at the University of Gothenburg, P.O. Box 465, SE-405 30 Gothenburg, Sweden; ‡ Department of Internal Medicine and Clinical Nutrition, Institute of Medicine, Sahlgrenska Academy, University of Gothenburg, P.O. Box 459, SE-405 30 Gothenburg, Sweden

## Abstract

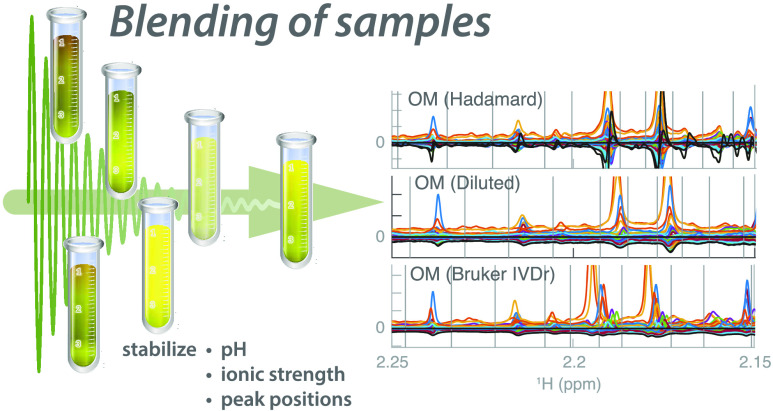

Urine is an equally attractive biofluid for metabolomics analysis, as it is a challenging matrix analytically. Accurate urine metabolite concentration estimates by Nuclear Magnetic Resonance (NMR) are hampered by pH and ionic strength differences between samples, resulting in large peak shift variability. Here we show that calculating the spectra of original samples from mixtures of samples using linear algebra reduces the shift problems and makes various error estimates possible. Since the use of two-dimensional (2D) NMR to confirm metabolite annotations is effectively impossible to employ on every sample of large sample sets, stabilization of metabolite peak positions increases the confidence in identifying metabolites, avoiding the pitfall of oranges-to-apples comparisons.

## Introduction

Liquid NMR is a relatively insensitive but robust technique, making it possible to record and reproduce spectra accurately with intensities proportional to molecule concentrations. These can be determined with a precision of less than micro molar in noncrowded spectra with few molecule types from smaller sample sets where the time consumption on the instrument is not limiting. In metabolomics, this is rarely the case though. In practice, large sets of samples require shorter experimental time per sample hampering precision. And worse still, complex molecular mixes, like urine where pH and ionic strength vary in a wide range, give crowded spectra where the resulting peak shift variability makes interpretation difficult and possibly inaccurate due to between-spectra intensity misalignment or misassignment. This inaccuracy problem can to some degree be solved by using Diluted samples, reducing peak shift inconsistencies between spectra but at the price of reduced precision in estimating metabolite concentrations. Modern cryo probes exhibiting increased sensitivity can extend the detection limit to lower concentrations, but the shift problem when peaks overlap remains. Two dimensional spectra can resolve overlap situations but, similar to the approach of diluting samples, has the drawback of sensitivity loss. Therefore, measuring crowded one-dimensional (1D) urine spectra with in between shift inconsistencies is still common practice and motivates the use of sophisticated automatic or semiautomatic software to handle analysis. These approaches rely on deconvolution of spectra matched with spectral database information on relevant metabolites at the relevant temperature, pH and ionic strength (if known), to determine the corresponding concentrations. This is available through commercial (Bruker IVDr; Nightingale Health; ChenomX Inc.) as well as academic tools/initiatives.^1−4^ The high signal density in urine 1D ^1^H spectra usually results in that these targeted approaches leave major fractions of the signal unexplained, however, and the remaining parts are analyzed using simpler untargeted approaches like bucketing, or peak picking followed by alignment,^5^ in conjunction with correlation tools like STOCSY.^6^ For a recent review on ^1^H NMR metabolomics, see Vignoli et al.^7^

Since both the targeted and untargeted approaches have limitations due to the complexity of the spectra, the present work intends to investigate if there is a possible workaround to improve NMR spectral interpretation by measuring mixed samples. Mixing urine samples leads to an averaged sample pH and ionic strength and therefore also less peak shift variability. We demonstrate such an approach and back-calculate the spectra of original samples in a urine data set from a crossover three-meal breakfast study where the metabolic response of vegan (VE), lacto-ovo vegetarian (LOV), and omnivore (OM) breakfast challenges were conducted in healthy individuals, and the sampling was performed both pre- and postprandially. For comparison, the experiments were repeated three times on traditionally prepared samples, Diluted samples, and samples from each breakfast mixed separately according to a Hadamard matrix prior to measurement. Biological interpretation of the outcome of this particular project will be published elsewhere (Lindqvist et al., in preparation).

In the present work, we show that the proposed methodology is a viable alternative addressing the problems of matrix variability preacquisition rather than at the data analysis stage, provides error estimates, is automatable, and avoids the danger of comparing apples to oranges as can be the case for binned data in urine ^1^H NMR metabolomics.

## Theory

Without considering pH and ionic strength effects, it is in principle possible to reconstruct NMR spectra from a full Hadamard factorial design scheme of sample mix spectra since the NMR response is linear to concentration changes. The idea is that instead of having one measured spectrum per original unmixed sample, every measurement should add or subtract to the potential spectrum of a given original sample by having fractions of each sample in half of all measurements in various combinations orthogonal to each other. The original sample spectra are calculated using a Hadamard transform starting from the acquired spectra that display smaller pH and ionic strength effects than in the traditional approach. A Hadamard transform is performed by multiplying a set of data with a Hadamard matrix. For example, from four traditionally recorded spectra *x*1–*x*4
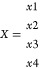
and the 4 × 4 Hadamard matrix
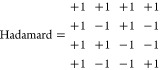
it is possible to obtain something we denote Hadamard spectra
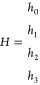
via the matrix multiplication
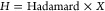
1This operation has limited value, however, since *H* has little value in its own, it is in principle possible to transform the corresponding metadata too and do the analysis in the Hadamard space. But, each row *i* from the second to the last in *H* can instead be measured in two measurements by calculating the difference between an NMR spectrum *h*_pos*i*_ of mixtures of all samples with a corresponding “+1” and an NMR spectrum *h*_neg*i*_ of mixtures of all samples with a corresponding “–1” in the Hadamard matrix, i.e.
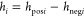
2The sum *s_i_* of the two spectra in each pair can be used for quality assurance since this sum should be equal for all spectra pairs, i.e., *s*_*i*_ = *s*_*j*_ in
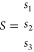

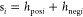
3The first Hadamard spectrum *h*_0_ can be calculated as the average of all *s*_*i*_
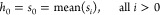
4For example, to obtain *h*_1_, the first and third, and the second and fourth samples are mixed and measured separately and the first spectrum is subtracted with the second. To obtain *h*_0_, the mean value of all six measured spectra is calculated and multiplied by two. Since the Hadamard matrix is orthonormal, it is also possible to calculate *X* from *H*
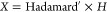
5where *X* is constructed spectra for each individual sample as it would have been without mixing but with smaller peak shift deviations than if measured directly. It might seem that it is not necessary to record both *H*_pos_ and *H*_neg_ since it is possible to derive one from the other if calculating *h*_0_ only from one of them and using that each pair is mirrored in *h*_0_/2. But if both *H*_pos_ and *H*_neg_ are not measured, the possibility for accuracy and precision estimates in the Hadamard space is lost while the use of both, i.e., an overdetermined system, allows for calculating *S* and checking the consistency. The combined shift effect when changing both pH and ionic strengths is not necessarily monotonic as the Henderson–Hasselbalch equation, but in practice, peaks shifted down on the ppm axis in *h*_pos*i*_ will most likely be shifted up in *h*_neg*i*_, and vice versa. This can be used when annotating spectral features to specific metabolites.

*h*_0_ is a measured value with a measurement error. This error is avoided by using mean value subtracted versions *H*_, *S*_, and *X*_ instead of *H*, *S*, and *X*, where the first row *h*_0_ is replaced with zeros in *H*, the mean value of all rows in *S* is subtracted from *S*, and *X*_ is calculated using *H*_ instead of *H*. If *X* is needed, *h*_0_ can be added to *X*_. This highlights the conceptual difference between conventional experiments and their Hadamard versions. In conventional experiments, the error in the measurements and after interpretation is individual and related to zero. In the Hadamard experiment, errors are smeared over all calculated NMR spectra independent of peak sizes and relate to the difference to the mean intensity rather than zero.

Often, it is more relevant comparing a particular measurement with the mean of the group rather than zero, as is done, for example, in principal component analysis (PCA). But sometimes absolute values are sought. Also, there is always a risk of batch effects correlated to sample order. This justifies replacing some samples with sample blanks in the Hadamard setup to better estimate how accurate and precise zero intensity is determined. Assuming that any batch effect is changing smoothly over the experimental run, differences between blanks placed in the matrix with +1 and −1 equally distributed over the experiment will describe the overall precision, with batch effects not counted. Differences between blanks placed in the matrix with +1 and −1 unevenly distributed over the experiment will also show any accuracy problems due to sample order.

Measurement errors are usually considered less severe if they reduce precision, possibly giving rise to false negatives, and worse if they are biased, giving rise to false positives. *S*_ spans the mean value subtracted Hadamard space and can be projected onto a subspace of extra relevance, for example, a vector discriminating two groups in an Orthogonal Partial Least Squares Discriminant Analysis (OPLS-DA). In the small example above, if *s*__2_ = *s*__3_ ≈ 0 but *s*__1_ differs, the former two support each other suggesting that OPLS-DA models of sample 1 and 2 vs 3 and 4 or 1 and 4 vs 2 and 3 are more likely fine whereas 1 and 3 vs 2 and 4 must be examined carefully.

## Experimental Section

### Ethical Approval, Participant Recruitment, and Study Design

The project adhered to the Helsinki Declaration and was granted ethical approval by the Regional Ethical Review Board in Gothenburg (reference number 561-12). The study was registered with ClinicalTrials.gov (identifier: NCT02039596). In short, in a crossover design, each study participant consumed a vegan (VE), lacto-ovo vegetarian (LOV), and omnivorous (OM) breakfast in a randomized fashion during three consecutive days, after overnight fasting. All 32 volunteers had a VE breakfast. One dropout led to LOV and OM breakfasts being eaten by 31 participants.

### Sample Collection and Preanalytical Handling

Sampling was performed as described in Lindqvist et al. (in preparation). Briefly, urine samples were collected pre- and 3 h postprandial the breakfast meals, respectively. In total, 188 samples were collected. Samples were spun at 3800 rpm at 4 °C for 10 min before taking aliquots of the supernatant. Samples were stored at −80 °C until analysis. Before NMR sample preparation, samples were thawed at RT for 2 h and subsequently centrifuged at 4 °C and 2000 rpm for 5 min.

### Bruker IVDr Sample Preparation

70 μL of urine buffer (1.5 M KH_2_PO_4_ pD 6.95, 0.1% TSPd_4_, 0.5% NaN_3_ in 99.8% D_2_O) was transferred to each well of a deepwell plate (DWP) using an Eppendorf E3x multipette. 630 μL of urine was transferred to the DWP containing buffer using a SamplePro Tube L liquid handling robot (Bruker BioSpin). The DWP (samples referred to as “Bruker IVDr”) was subsequently shaken at 12 °C, 800 rpm for 1 min, and briefly centrifuged at 4 °C at 3700 rpm for 1 min in a swing-out rotor. 600 μL from each DWP well was transferred into 5 mm SampleJet NMR tubes (Bruker BioSpin) using the SamplePro.

### Hadamard and Diluted Sample Preparation

A joint source DWP was generated for the Diluted and Hadamard sample matrices, one breakfast at a time, on three occasions. Using an Eppendorf E3x multipette, 190 μL of urine buffer was transferred to 64 wells of the source DWP (equal number of samples from one breakfast occasion). Subsequently, 1700 μL of urine of each original sample was manually pipetted into the source DWP which was then sealed with a polymerase chain reaction (PCR) plate film, shaken at 400 rpm for 5 min at 10 °C, and then centrifuged at 2000*g* for 1 min at 4 °C. 161 μL of each source DWP well was transferred to a new DWP (“diluted”) and was filled with 490 μL 90:10 water/urine buffer in each well with a Bravo liquid handling robot (Agilent). 161 μL sample from the source plate was added to the Diluted DWP with the Bravo, shaken, centrifuged, and samples transferred to 5 mm SampleJet tubes as for the Bruker IVDr samples described above. The Hadamard VE, LOV, and OM mixing designs were generated using an Eppendorf E3x multipette. 25 μL of each urine sample from the source DWP was distributed to, in total, 63 wells of two new DWPs (“Hadamard positive” and “Hadamard negative”) according to a dispense order defined by the Hadamard matrix design (available upon request). For 64 original samples, the number of dispense steps was in total 4032 for each breakfast. For the LOV and OM breakfasts, samples from one individual was missing, i.e., there were samples from 31 individuals compared to the VE breakfast including samples from all (*n* = 32) individuals. Missing samples from LOV were substituted with a water solution containing 3.81 mM fumarate and OM water only. The original samples were arranged so that all preprandial samples were measured in *h*_pos1_ and all postprandial samples were measured in *h*_neg1_. After completing all dispense steps, the Hadamard DWPs were sealed, shaken, centrifuged, and samples transferred to NMR tubes as described above for Bruker IVDr and Diluted samples. All samples were kept cold during preparation and prior to analysis. For an overview of the different sample preparation protocols employed, see Figure S1.

### NMR Experiment Data Acquisition and Processing

All spectra were acquired on a Bruker 600 MHz Avance III HD spectrometer according to the Bruker IVDr SOP. For details, see the Supporting Information.

### NMR Data Analysis

**Figure 1:**
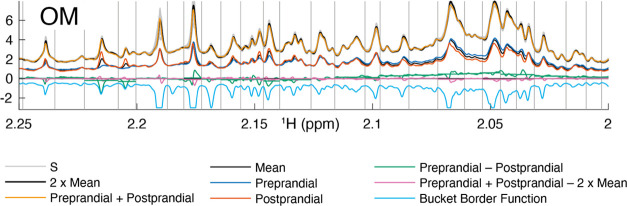
OM breakfast Hadamard example. The *H*_pos_ and *H*_neg_ spectra are the starting points in the calculations. For clarity, only the first out of 63 *H*_pos_–*H*_neg_-pairs for the OM breakfast is shown, *h*_pos1_ (blue) and *h*_neg1_ (red). Since all preprandial samples are mixed in the *h*_pos1_ sample and their corresponding postprandial samples in the *h*_neg1_ sample, they are labeled accordingly. *S* (*H*_pos_ + *H*_neg_), are drawn in thin gray, except for the first pair *s*1 (*h*_pos1_ + *h*_neg1_) which is drawn in thick yellow. *h*_0_ (the mean of all *S*) and *h*_0_/2 are drawn in thick and thin black, respectively. All pairs (rows) in *S* should, in theory, overlap each other and *h*_0_ since they should be the same. *h*_pos1_ and *h*_neg1_ should preferably deviate equally much but with opposite signs from those of *h*_0_/2. Consequently, *s*_1 (purple) should be zero and a deviation tells that spectra do not match locally. *h*_1_ (green, *h*_pos1_ – *h*_neg1_) is the intensity which, using the Hadamard transform, will add or subtract equal amounts of intensity to all prandial or postprandial samples, respectively. A simple bucketing algorithm finds local minima of the three breakfasts’ summed standard deviations std(*S*_) + std(*H*) (light blue, mirrored in the *x*-axis in the figure) used as bucket borders (vertical gray lines). As seen for *s*_1 compared to its bucketed version (both purple), a lot of the discrepancies disappear when integrating the buckets. Remaining differences in *S* and *s*_*i* can be used for general and preprandial–postprandial error estimates. Figure S3 shows the same region for all breakfasts.

All 1D nuclear Overhauser effect spectroscopy (NOESY) spectra were imported to Matlab (R2019b, MathWorks Inc.) using rbnmr.m^8^ and normalized to their integral of the ERETIC peak. In addition, in the Hadamard approach, the spectra were normalized setting the cumulated sum of each pair *s_i_* = *h*_pos*i*_ + *h*_neg*i*_ between 0.1 and 4.1 ppm to equal sum (Figure S2). *H*_, *S*_, and *X*_ were calculated. Data was bucketed setting bucket borders where the sum of the standard deviations of *S*_ and the standard deviations of *H*_ for the three breakfasts was at a local minimum, not larger than 3 × 10^–5^ in intensity or closer than 40 data points corresponding to 0.0061 ppm to another bucket border using the Matlab function islocalmin.m (Figures 1 and S3). Bucketing of the Bruker IVDr and Diluted spectra was performed in the same way but using local minima of the standard deviation of the spectra plus the spectra mean values as bucket borders. Hadamard-transformed VE, LOV, and OM spectra *X* were calculated from their respective *X*_ and *h*_0_. All Bruker IVDr, Diluted, and Hadamard spectra were normalized to their integral between 0.1 and 4.1 ppm (Figure S4). The Bruker and Diluted approaches used the full −0.1 to 9.5 ppm range, whereas for Hadamard, the ppm ranges 0.11–2.95, 3.10–3.27, 3.32–3.99, 5.20–5.51, 6.00–7.04, 7.09–7.13, 7.24–7.48, 7.53–7.69, 7.72–7.82, 7.90–7.93, 8.01–8.02, and 8.50–9.50 were used, and all other parts of the spectra were excluded due to *s*_1 describing the postprandial vs preprandial differences dimension being clearly off zero for at least one breakfast. Standard multivariate analysis was performed independently on the Hadamard, Diluted, and Bruker IVDr data using Simca version 15.0.2 (Sartorius Stedim). Principal component analysis (PCA) was used for outlier detection. Orthogonal Partial Least Squares Discriminate Analysis (OPLS-DA) models were used to verify metabolic postprandial vs preprandial changes for each breakfast. Orthogonal Partial Least Squares Effect Projection (OPLS-EP),^9^ where the effect matrices were the postprandial data subtracted with the preprandial data for each person, was used for identifying the metabolic response of each breakfast with the loadings describing the response and the cross validated standard errors of their variability. The effect matrices were also used in the OPLS-DA models to highlight differences between the meals. Univariate scaling was applied to all of the buckets. All OPLS-DA and OPLS-EP models used seven cross-validation groups with samples from four different persons in each group. Autofit was used to determine the number of significant components.

Metabolite annotations were made manually using ChenomX (Chenomx Inc.) and the Human Metabolome Database on the 1D NOESY spectra, with additional help from 2D natural abundance ^1^H,^13^C-HSQC, 2D J-resolved, and ^1^H,^1^H-TOCSY spectra of the *h*_pos1_ and *h*_neg1_ Hadamard samples. Experimental details for the 2D measurements are available upon request.

## Results and Discussion

The spectra pair sums *s*_*i*_ have intensity biases, motivating the extra normalization step of the Hadamard spectra. The biases should not be related to the NMR measurement since spectra were normalized to the ERETIC peak but could possibly reflect inevitable pipetting errors when mixing samples (Figure S2).

As expected, the pairs show very similar s_*i*_ values after the normalization. Differences are mainly due to peak shifts rather than intensity inconsistencies (Figures 1 and S3) with three exceptions for urea (Figure S7), water, and TSPd_4_. The latter can be explained by particular features of each measured experiment, i.e., differing pH, water suppression, and TSPd_4_ concentration. The LOV data set had a pH bias between the 2 days it was measured, and identical water suppression and perfectly consistent manual pipetting for that number of pipetting steps were difficult. There was an unfortunate shimming problem when recording the Hadamard spectra, in particular when projected onto the relevant postprandial vs preprandial dimension which resulted in that we in the final Hadamard data set left out data around water between 4 and 5.2 ppm and creatinine 2.96 and 3.1 ppm due to poor *S*_ consistency. This would have been much less problematic if we had randomized the samples in the Hadamard matrix, but for illustrative purposes (as shown in Figure 1), we chose to place them so that we would have one Hadamard spectrum with all preprandial and one with all postprandial samples, unnecessarily transforming increased variability to an accuracy error. However, the advantage with the Hadamard approach is that this error can be quantified using *s*__1_ (Figures 1 and S3).

The Hadamard mixing of samples even out ion concentration and pH differences, but the spectra still suffer from smaller phase shifts making direct comparison between spectra less attractive. A natural choice would be using a targeted approach and deconvolution software like ChenomX but that would introduce a subjectivity and increased complexity which we, in this case, wanted to avoid. Instead, we chose to use bucketing, which is robust and also allows the use of all data rather than only selected parts and without the need for annotation. When buckets are used, the positioning of the borders is important. The aim is to have all of the intensity from corresponding peaks from various spectra within the same bucket, without having too many different peaks representing different molecules within the same bucket. The former often requires larger bucket widths and the latter smaller, so some compromise is needed where peaks are cut and present in neighboring buckets at the same time as a given bucket is defined by contributions from more than one molecule. In practice, the quality of the bucketing is often less satisfying due to bad alignment, since different proportions of peak intensities in the buckets from different samples will give too high or too low concentration estimates. The approach we used, determining bucket borders in the Hadamard spectra, avoiding variations of *S*_ indicating local peak shift inconsistencies, or variations in *H*_ the presence of a peak, was relatively robust with few places where manual intervention could have been justified after setting some reasonable number for maximal standard deviation and minimal bucket size (Figures 1 and S3). At ppm values larger than 7 ppm, peak shifts were often too large, however, resulting in inconsistencies in *S*_ and subsequent need for removal after manual inspection in the final analysis. We could have kept most of these if we would have allowed ourselves to manually merge buckets, but this time, we decided to avoid any user intervention. The similar bucketing approach used on the spectra of the Diluted samples worked surprisingly well compared to where we manually from visual inspection put bucket borders. Both the Hadamard and Diluted buckets suffered slightly; from that, the data for the different breakfasts were obtained with significant time in between, resulting in some ppm-shift batch effects. Setting bucket borders in the Bruker IVDr spectra without alignment often seemed like an impossible task independent of method, but we cannot see that we could have done it better manually.

**Figure 2:**
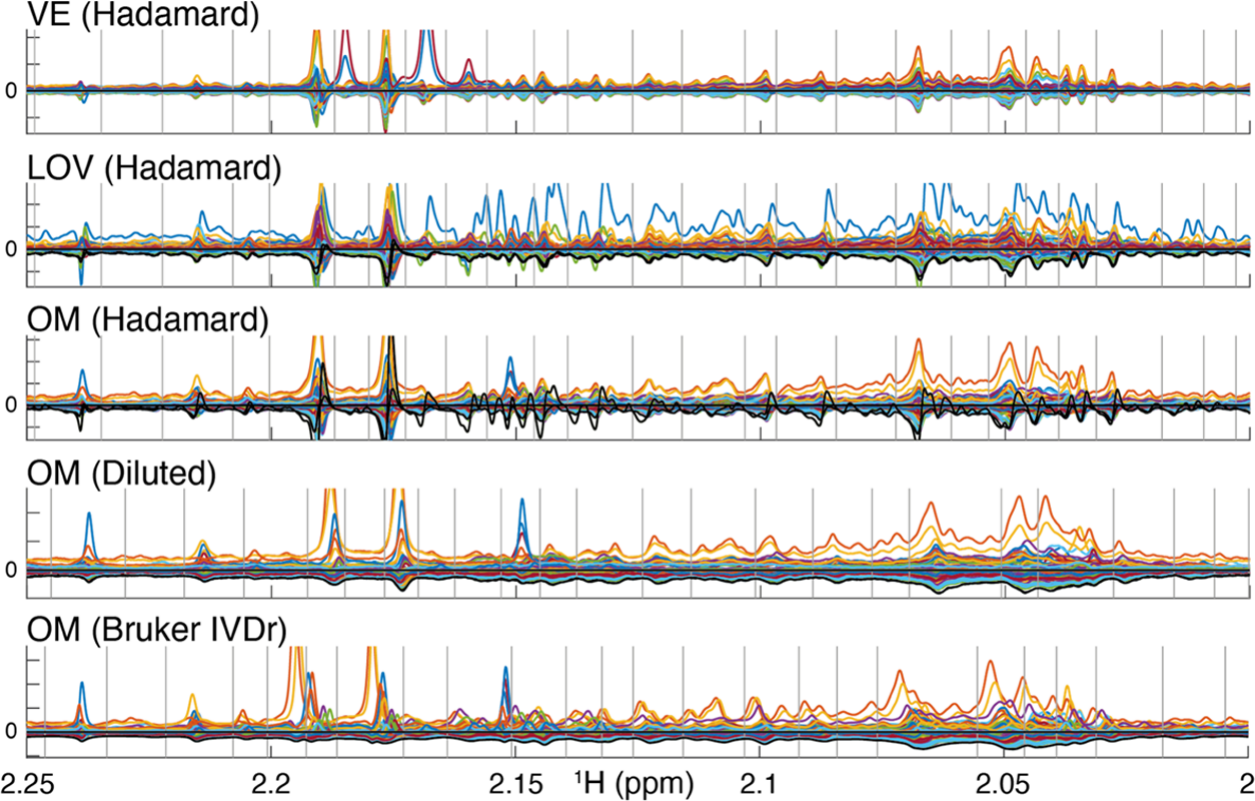
Each plot shows an overlay of the calculated Hadamard spectra and Diluted and Bruker IVDr spectra in the same region as in Figure 1 with a separate color for every sample. Mean subtracted versions are shown corresponding to *X* from the Hadamard calculation. The breakfast and sample preparation methods are indicated in the panel headings. The VE Hadamard setup did not include any sample blanks. LOV and OM Hadamard setups included two blanks (black). The Bruker IVDr setups did not include sample blank data acquisition; instead, the black line in the Bruker IVDr OM panel is the negative of the mean. The VE Hadamard do not show any obvious day-to day (*h*_pos_ vs *h*_neg_) batch effect while the LOV does, resulting in a bad calculated sample 1 with apparent but incorrect high intensity (blue). Both of the LOV Hadamard blanks placed on rows 47 and 48 in the Hadamard matrix correspond to rapid experimental Hadamard sample to sample inconsistencies that can be described as noise or reflecting the precision of the calculated spectra. They show generally good in between similarity and also have the smallest bucketed intensity compared to the other LOV since they are devoid of peaks in the region. The Hadamard LOV sample 1 includes the worst part of the batch effect, while the others mostly lose resolution. The blank samples of the OM Hadamard were placed in rows 1 and 2, where row 1 (black with wiggles) shows most of the day-to-day batch effect which unfortunately also was present in the OM. The row 2 blank calculated spectrum (also black) smoothly runs slightly below the others, indicating the precision, not including the batch effect absorbed by the other blank. Spectra are similar, independent of sample preparation method (only OM shown). The Hadamard peaks are generally sharp. When there is bad alignment despite the mixing of samples, e.g., at 2.18 and 2.19 ppm, the peaks get strange shapes but their integrals are fine if the buckets are wide enough covering the corresponding measured Hadamard peaks. The Diluted spectra are relatively well aligned as well, while the Bruker IVDr spectra have the best signal-to-noise ratio but are difficult to interpret due to less good alignment. The most concentrated sample is approximately nine times stronger than the least, which is challenging for all methods. Also, after normalization, PCA showed clear concentration trends (not shown), but fortunately the preprandial and postprandial breakfast pairs had similar overall concentration, which should reduce errors in the present case.

Direct comparison of the three types of spectra prior to bucketing gives similar results but with differences in the details. The Hadamard calculated spectra show distinct variability along the ppm axis. Minor misalignment exists in the Hadamard spectra but is only severe above 7 ppm. Misalignment results in skewed peaks in the calculated spectra but correct bucket intensity when misalignments stay within the buckets. The corresponding mean value subtracted versions of the Diluted sample spectra have slight peak shift variations. Since some of the samples are very dilute, some peaks have very low signal-to-noise. Bruker IVDr spectra have superior signal-to-noise but more peak shift variability (Figure 2).

One person dropped out, and the corresponding two sample positions in the LOV and OM breakfasts were replaced with sample blanks. In the LOV sample set, these were placed on rows 47 and 48 in the 64 × 64 Hadamard matrix with corresponding added and subtracted spectra distributed relatively evenly over the entire experiment. Their in between variability looks like noise, and the approach seems good for precision estimates. In the OM sample set, the blank samples were placed on rows 1 and 2. The Hadamard calculated spectrum of row 1 had all added Hadamard spectra recorded 1 day and all subtracted another day, catching common between day batch effect accuracy problems, leaving the others almost free from this since they were all recorded in half of the experiments on both days. Properly arranged sample blanks can be used as quality controls obtained at the same time as the measured samples rather than in between samples as is the norm (Figure 2).

To check how much unwanted in between calculated sample spectra crossover the Hadamard approach gives in practice due to imperfect pipetting, not absolutely consistent NMR data etc., concentrated fumarate was added in the LOV blank sample resulting in a large peak at 6.52 ppm, a region which otherwise is almost free of signal in human urine (Figure S5). The corresponding VE Hadamard calculated and Diluted bucket has good resemblance and correlated intensity variation close to noise, picked up also by Hadamard. For LOV, the additions and subtractions of the fumarate peak has not completely canceled out, and the Hadamard calculated spectra therefore show larger variability than the corresponding Diluted data. A closer inspection show that this mainly has affected the samples on rows 1, 33, 17, and 49 in the Hadamard matrix which are most prone to batch effects. The Hadamard LOV experiment was unfortunate in the sense that there is a large day-to day variation as seen for example in urea (Figure S7). Still, the vast majority of the Hadamard calculated spectra has a variability not much wider than the Diluted which must be considered good since measuring signals close to zero intensity and comparing these with absolute zero intensity rather than with the mean of spectra are not ideal for Hadamard in a matrix also including very large peaks. Using sample blanks at Hadamard positions risking batch effects, in this case at 1, 33, 17, and 49, to clean the remaining data from that, in combination with randomizing the experimental sample order within each such segment, in this case *h*_pos_ and *h*_neg_ 2–16, 18–32, 34–48, and 50–64, should give even less crossover. Also, since the sample blanks only dilute the spectra, it should be possible to replace explicit water samples with corresponding decreases in spectrum intensity, but this has not been tested.

Integral normalization was used to get an objective normalization without the influence of any peak interpretations as it would have been after bucketing using, e.g., PQ-normalization. There is good agreement between the Diluted, Bruker IVDr, and Hadamard calculated spectra in terms of relative intensity. The Hadamard intensities do not stick out compared to the other two groups. The least concentrated samples are approximately nine times more diluted compared to the most concentrated. Despite being taken at different times of the day, the pre- and postprandial samples are relatively equally diluted reducing the risk of accidently interpreting concentration differences as before vs after breakfast differences (Figure S4).

The Diluted and Hadamard breakfast samples had, for practical reasons, to be analyzed one breakfast at a time, a long time apart. OPLS-DA models of the preprandial data show no trends between the breakfasts (data not shown). Therefore, in combination with that, we are only considering the postprandial subtracted with the preprandial effect matrices; there should not be any risk of bias between the data of the different breakfast types in this respect.

PCA models on all three sets of data and also the relatively concentrated Bruker IVDr data showed that at least one sample from three different participants was a clear outlier. The reason was due to low concentration; these also had the largest normalization constants. All samples from corresponding participants as well as the samples from the person who did not participate in all three breakfasts were excluded, leaving 28 persons. PCA models of the remaining data showed a trend correlated to concentration, which the integral normalization had not taken care of fully. Since each person’s preprandial–postprandial sample pair had relatively similar concentration, this was not considered a problem.

**Figure 3:**
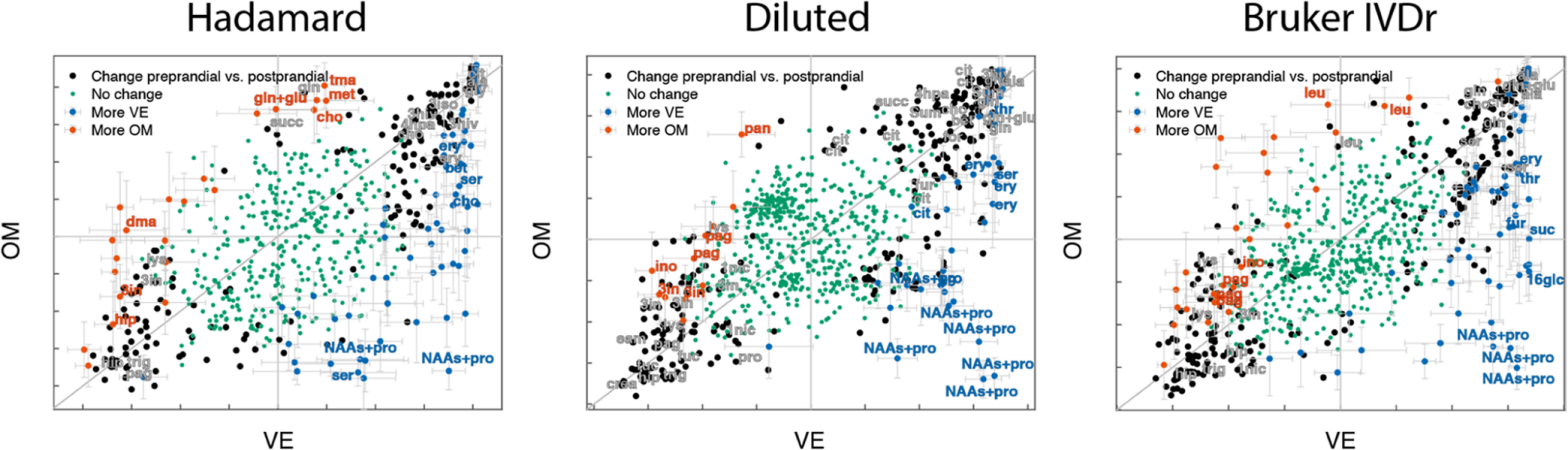
Figures show the loadings with cross validated standard errors from the VE OPLS-EP model vs the loadings with cross validated standard errors from the OM OPLS-EP model using Hadamard, Diluted, and Bruker IVDr data. The black color code describes buckets with loading values larger than corresponding cross validated standard errors in either the VE or OM model but without significant VE vs OM differences. The blue and red color codes describe the corresponding bucket values from VE vs OM OPLS-DA models of the effect matrix data, blue being significantly higher in VE and red in OM defined as the size of loading values being larger than corresponding cross validated standard errors. The first quadrant has bucket intensities corresponding to metabolites generally increasing after both the VE and OM meals. Of note is that some loadings are colored blue in the upper right corner meaning that despite a large increase both after VE and OM meals, the increase is larger after VE. The second and fourth quadrants show that when some bucket intensities increase after OM, they decrease after VE and vice versa. The third quadrant show bucket intensities decreasing after both VE and OM meals. Of note is that some loadings are colored red meaning that the decrease is lower after the OM meal. It might seem contradictory that some blue colored bucket intensities are above the diagonal. It must be remembered that the position of the loadings describes the relative concentration changes after each breakfast while the colors better represent if a particular bucket intensity or metabolite concentration increase or decrease more depending on breakfast type. The most striking fact when comparing the results from the three types of analysis is that the final results are both qualitatively and quantitatively very similar. None sticks out compared to the other in any particular way. A few selected buckets are denoted by abbreviated names (Table S1).

As expected, there are large responses in all of the postprandial vs preprandial OPLS-DA models with high *Q*^2^ values well above permutations (Table 1) and scores clustering well off zero for all participants (not shown). The corresponding postprandial–preprandial OPLS-EP model loadings and their cross validated standard errors were used to describe metabolomic changes for each breakfast. OPLS-DA models of pairs of breakfast types on the effect matrices were used to quantify the difference between breakfasts as classification performance. There are a lot of similarities in the responses between the breakfast types, and the differences are less pronounced (Figures 3 and S6, Table S1). However, VE deviates clearly from both the LOV and OM breakfasts (Table 1), with all participants’ breakfasts always correctly classified (not shown). The LOV and OM difference is more subtle, as reflected in *Q*^2^ values (Table 1). In most cases, Hadamard, Diluted and Bruker IVDr protocols perform very similar in terms of *Q*^2^ values. A difference is that the Hadamard OPLS-DA model of the VE vs LOV breakfasts is good, but slightly worse than the other two, while the Hadamard OPLS-DA model of LOV vs OM is less good but clearly better than the others. To check the robustness of the models, OPLS-DA models of LOV and OM of the 14 first persons were used to predict the breakfasts of the last 14 persons, and vice versa. A correct prediction was defined as having the correct sign of the score, and the results supported the higher Hadamard *Q*^2^ value. 51 out of 56 predictions were correct when using the Hadamard, 43 when using the Bruker IVDr, and 44 when using the Diluted protocol, respectively (not shown).

**Table 1:**
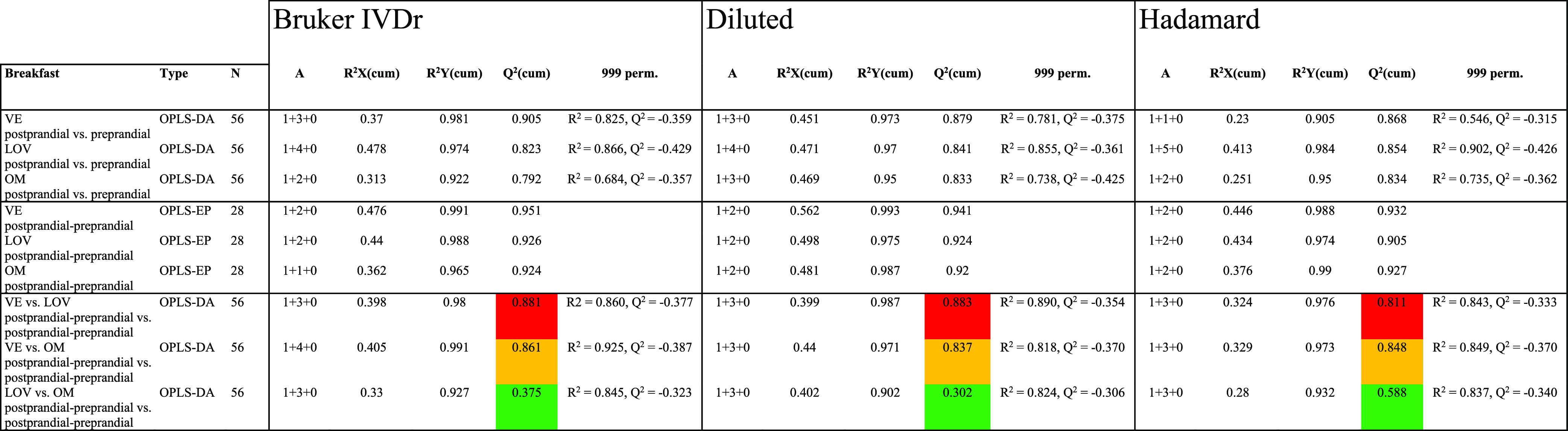
OPLS-DA and OPLS-EP Model Classification Performance

The Hadamard data set has fewer buckets than the corresponding Diluted and Bruker IVDr data sets since in the former, a large fraction was excluded due to bad water suppression or remaining peak shifts downfield from 7 ppm, caught in the manual *S*_ and *s*__1_ evaluation. Using a similar set of buckets also for Diluted and Bruker IVDr, for consistency, only changes these models marginally with *Q*^2^ values increasing or decreasing not more than about one percent (not shown). It is difficult to see why some method performs slightly better in terms of separation in one model but worse in another. A speculation is that it has to do with which buckets are most important where low intensity peaks with little shift variability benefits the Bruker IVDr method while larger peaks and larger shift variability are relatively better for the Diluted and Hadamard approaches. Metabolites being important in the various models are not only qualitatively, but generally also quantitatively very similar, independent of method, if comparing with the internal uncertainties expressed as for example the cross validated standard errors.

In terms of annotation, Hadamard outcompetes the other two methods in that the peak position confidence is much higher. Since the peaks are both high in intensity and do not move much the analysis could to a large extent be done straightforward from visual inspection of overlaid 1D spectra as in Figures 1 and 2, in combination with STOCSY.^6^

Only a subset of bins was possible to annotate to specific metabolites, in line with the overall difficulty in NMR urine metabolomics of identifying metabolites judging from chemical shift alone or without discernible intensity in ^1^H–^13^C natural abundance correlation spectra. Among the annotated metabolites, alanine, glutamine, glutamate, choline, and tyrosine increased the most postprandially, independent of breakfast, while creatinine, trigonelline, hippurate, and *N*-phenylacetylglycine were among those decreasing the most. *N*-acetylated amino acids and proline showed a larger increase in VE than in LOV and OM, while leucine showed a larger increase in LOV and OM compared to VE. Although the trends are less pronounced comparing LOV and OM, there are no annotated metabolites consistently enriched/depleted across the three sample preparation protocols (Figure S6, Table S1).

## Conclusions

Despite the lab work increase due to demanding manual pipetting, the Hadamard design overall performed at least as well as the other two more conventional methods in terms of discriminating postprandial from preprandial samples and one breakfast from the other. Consistent pipetting over many hours is difficult and tiresome, and it was obvious that a liquid handling robot would perform mixing not only much faster but also better in terms of precision. We used 64 samples but a robot would be able to pipet more than that, e.g., 128 or 256, in a day and realistically without precision losses. This will be investigated in the future. If it is possible using 256 samples, only very little shift variability should remain which in practice often is what is most resolution-limiting and not, e.g., the magnetic field strength.

The increase in complexity in the lab pays off when analyzing and annotating the spectra since the peak shifts are very consistent and the use of more spectra than samples allowed evaluation of the quality of each bucket individually. It would be interesting to see if the contemporary use of metabolite databases in combination with deconvolution to assign peaks can take advantage and perform better by using Hadamard data with its extra constraints and improved peak consistency.

Similar to what others have reported,^10^ our analysis indicate that it would be fruitful both for traditional measurements and the Hadamard approach to dilute only the stronger but not the weaker samples in order to even out concentration differences before measurements. However, this would require some procedure to estimate concentration prior to NMR analysis, e.g., a specific gravity assay suitable for high throughput assessment (https://www.thermofisher.com/order/catalog/product/1194#/1194). It is worth noting that with the Hadamard approach, to gain sensitivity, it would be possible to add volumes of each original sample in each mix proportional to the inverse of its estimated concentration rather than diluting the most concentrated samples as long as the mix of samples had a sufficient volume. The weaker samples would dilute the stronger gain signal as well as decrease peak shift variability. Together with the use of a robot and maybe mixing 128 additions per sample, this should almost eliminate peak shifts variability in large parts of urine spectra.

It should also be said that we used the Hadamard matrix in our design of the experiment since it is perhaps the most common, but other matrices are worth considering as well. Setting each pair of *h*_pos*i*_ and *h*_neg*i*_ to be orthogonal to each other has the advantage of simplicity but using spectra where this is not true could potentially be advantageous. If some spectrum fails due to, for example, bad shimming, a group of others could then better cover up for the loss. The concept of using experimental designs measuring more spectra than original samples opens up not only for quantitative error estimates of concentrations and shift variability but also for quality control of lab work and magnet performance.

## Supporting Information Available

Hadamard sample preparation setup pseudocode; NMR experimental details; flowchart figure of sample preparation protocols; normalization figure; raw data example figure and OPLS-DA figures showing all three breakfast challenges and sample preparation protocols; figures of fumarate and urea over all sample sets; table of metabolites and associated abbreviations used in manuscript (PDF)
