# Supplementary material for: Blending Samples to Increase Accuracy and Precision of 1H NMR Urine Metabolomics
Source: Anal Chem. 2024 Jul 31;96(32):13078–85. doi: 10.1021/acs.analchem.4c01532 (PMC11325295; doi:10.1021/acs.analchem.4c01532)
Supplement: Supplementary file 1 — ac4c01532_si_001.pdf [file ac4c01532_si_001.pdf]

Supporting information

Blending samples to increase accuracy and precision of ^{1}H NMR urine metabolomics

Anders Bay Nord[#], Helen Lindqvist[§], Millie Rådjursöga[#], Anna Winkvist[§], B. Göran Karlsson[#], Daniel Malmodin[#]*

AUTHOR ADDRESSES: [#]Swedish NMR Centre at the University of Gothenburg, PO Box 465, SE-405 30, Gothenburg, Sweden; [§]Department of Internal Medicine and Clinical Nutrition, Institute of Medicine, Sahlgrenska Academy, University of Gothenburg, PO Box 459, SE-405 30, Gothenburg, Sweden

Table of contents

Hadamard sample preparation setup pseudo code

NMR data acquisition and processing

Figure S1.
Figure S2.
Figure S3.
Figure S4.
Figure S5.
Figure S6.
Figure S7.
Table S1.

Hadamard sample preparation setup pseudo code

1. Determine your number of urine samples (m).
2. Make a source plate with original urine samples:buffer, mixed 9:1.
3. Use a Hadamard matrix of size 2^n such that $2^{n-1} < m <= 2^n$, meaning that you will measure $2*(2^n-1)$ mixtures of m urine samples and 2^n-m 'blanks' (e.g. water or water spiked with some metabolites).
4. `Hadamard = hadamard(2^n)` Construct the Hadamard matrix e.g. by using Matlab.
5. `[mixture_pos, sample_pos] = find(Hadamard(2:end,:)+1)` Make a list how to pipette the samples into mixtures using the '+1' in the Hadamard matrix. Don't use the first row, instead determine the average spectrum of all samples from the mean of all spectra rather than measure it explicitly.
6. `[mixture_neg, sample_neg] = find(Hadamard(2:end,:)-1)` Make a list how to pipette the samples into mixtures using the '-1' in the Hadamard matrix.
7. Decide the order from 1 to 2^n you will pipette your blanks and urine samples. The samples will be mixed according to corresponding column in the Hadamard matrix as explicitly written in the lists.
    a. Distribute the blanks. Most important is to put blanks at columns in the Hadamard matrix with few sign changes to spot and remove any batch effects (e.g. 1, $2^{n-1}+1$, $2^{n-2}+1$, $2^{n-1}+2^{n-2}+1$..). To determine the 'noise' related to the pipetting and measuring and not depending on sample order you can put blanks at columns with many sign changes (e.g. 2^n, 2^{n-1}, 2^{n-2}, $2^{n-1}+2^{n-2}$ ..).
    b. Distribute the urine samples at the remaining positions. Most likely random order will work fine. To avoid bad luck, first 'pipette' the metadata by putting it in the planned sample run order and multiply it with rows 2 to 2^n of the Hadamard matrix. Aim for outputs resembling gaussian distributions rather than having single mixtures of very large importance compared to the others. (N.B! For illustrative purposes we did the exact opposite in the presented manuscript, mixing pre- and postprandial samples separately in one dimension.)
8. Mix samples by pipetting e.g. with a multipette.
    a. Pipette the '+1 mixtures', from 'sample_pos' to 'mixture_pos'. Use a step pipetter and aim for not having to refill (apart from the first sample needing one refill). E.g. if the Hadamard matrix is 64x64 use a pipette of 1ml and distribute 25µl in the respective mixtures.
    b. Pipette the '-1 mixtures', from 'sample_neg' to 'mixture_neg'.
9. Transfer mixtures to NMR sample tubes.
10. Acquire NMR data.
11. Check the spectra. Calculate the summed spectra. Normalize each pair of 'positive' and 'negative' spectra to have the same sum over a relevant region. The summed and normalized pairs should be very similar. If a particular pair in a particular spectral region deviates from the rest a decision must be taken if the spectrum region can be used which will depend on the research question at hand. 'Pipette' the metadata as described in 7b. If the result shows that the particular pair have little importance compared to the other pairs it is fine to keep that spectral

region. Otherwise, if it is obvious that one spectrum in the pair is bad due to e.g. bad shimming and it can be assumed the other is ok consider replacing the bad with the ok spectrum mirrored in the average of all spectra. Alternatively, remove the part of the spectrum entirely from the analysis.

12. Calculate mean value subtracted original spectra and original spectra by adding the mean value, of the original samples from the differences of the spectra pairs using the Hadamard transform.
13. Bucket or deconvolute the spectra of the mixtures.
14. Assign your buckets or deconvoluted peaks to specific metabolites.
15. Calculate the mean value subtracted intensities, and the intensities by adding the mean value, of the original samples from the spectra pair differences in bucket intensities or deconvolution intensities using the Hadamard transform.
16. Proceed as if the data was not obtained from mixed sample measurements, i.e. normalize, and analyze as you normally would.

NMR data acquisition and processing

A Bruker 600 MHz Avance III HD spectrometer equipped with a room temperature 5mm BBI probe and a cooled SampleJet sample changer was used. Samples were kept at 279K while in the SampleJet and at 300K during data acquisition. NMR data was acquired with the standard pulse sequence 'noesygppr1d' using parameter sets and QA procedures defined by the Bruker BioSpin IVDr manual. 1D NOESY experimental parameters were a spectral width of 20ppm, an acquisition time of 2.726s, a relaxation delay of 4s with collection of 32 scans into 64k data points. Zero-filling and exponential line broadening of 0.3Hz was applied prior to Fourier transformation, phasing and baseline correction. All spectral processing was automated by the AU program specified in the IVDr SOP, including referencing to $TSPd_4$ and addition of an electronic quantification signal (ERETIC) at 12ppm. All acquisition and initial processing of data was made in Topspin3.5pl7 (Bruker BioSpin). The Bruker IVDr spectra were recorded at one single occasion. All six samples from a given person were measured in randomized order immediately after each other. The different breakfast samples' Hadamard and Diluted spectra were measured at different times. Both Diluted samples for each breakfast for a given person were measured immediately after each other.

Figure S1. sample preparation flowchart. For Hadamard and Diluted sample preparation, a source DWP is generated from original urine samples and urine buffer. The source DWP then is used to prepare a Diluted DWP as well as for supplying sample for the experimental design of the two Hadamard DWPs, positive and negative. The Bruker IVDr SOP involves only mixing original urine and urine buffer in a 9:1 proportion before transfer to NMR tubes for acquisition.

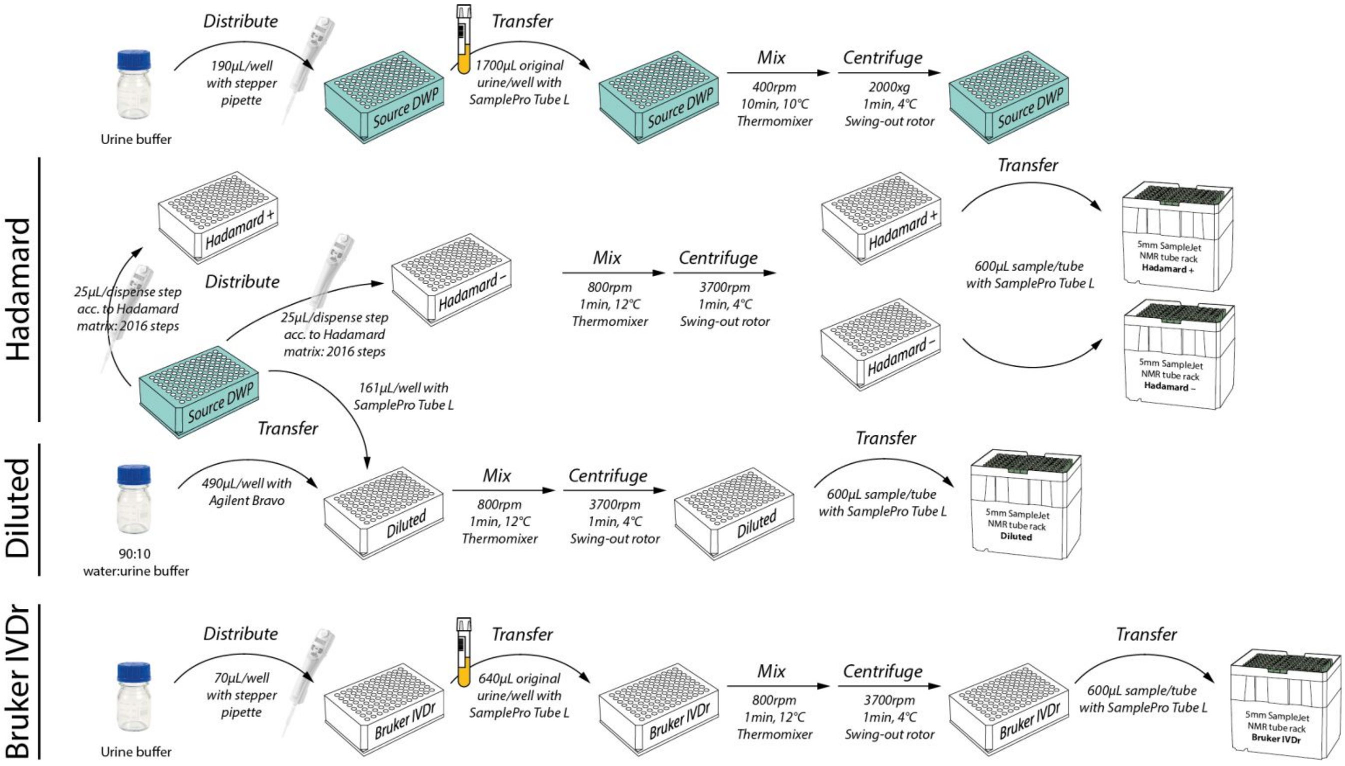

Figure S2. Normalization of Hadamard spectra. After normalization using the ERETIC signal, each spectrum pair, h_{posi} (blue) and h_{negi} (red), was multiplied with a constant, setting all integrals of h_{posi} + h_{negi} (grey) between 0.1ppm and 4.1ppm equal. Cumulated sum (A) before and (B) after setting integrals equal.

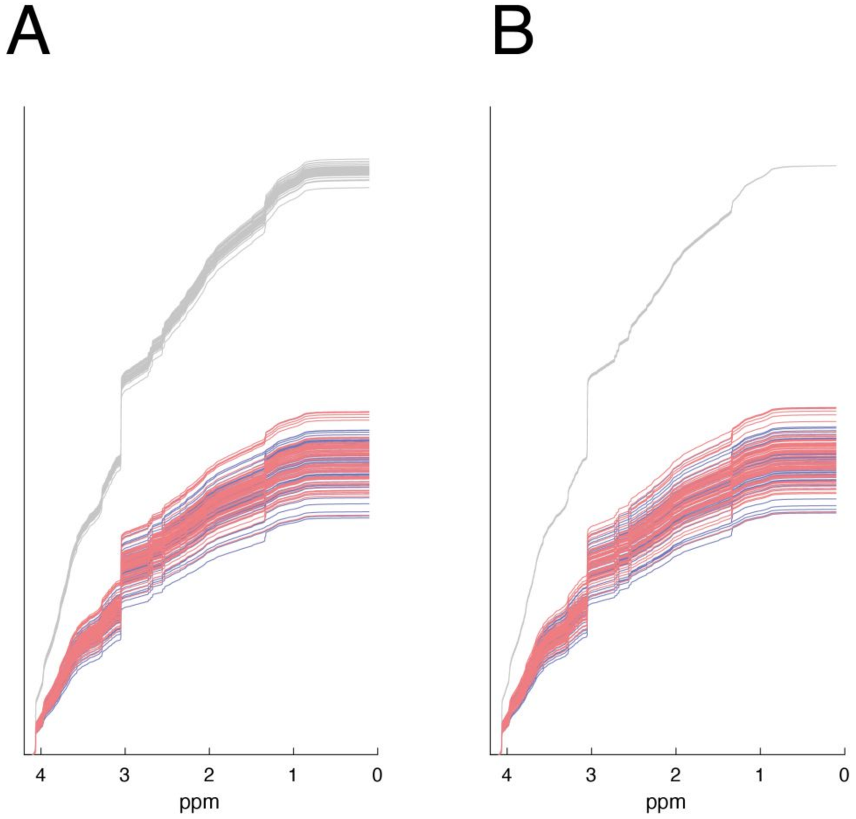

Figure S3: Hadamard examples. The H_{pos} and H_{neg} spectra are the starting point in the calculations. For clarity only the first out of 63 H_{pos}-H_{neg}-pairs for the OM breakfast is shown, h_{pos1} (blue) and h_{neg1} (red). Since all preprandial samples are mixed in the h_{pos1} sample and their corresponding postprandial samples in the h_{neg1} sample, they are labelled accordingly. S (H_{pos} + H_{neg}), are drawn in thin grey, except for the first pair s1 (h_{pos1} + h_{neg1}) which is drawn in thick yellow. h_0 (the mean of all S) and h_0 /2, are drawn in thick and thin black. All pairs (rows) in S should in theory overlap each other and h_0 since they should be the same. h_{pos1} and h_{neg1} should preferably deviate equally much but with opposite signs from h_0/2. Consequently, $s_{\_1}$ (purple) should be zero and a deviation tells that spectra don't match locally. h_1 (green, h_{pos1} - h_{neg1}) is the intensity which using the Hadamard transform will add or subtract equal amounts of intensity to all preprandial or postprandial samples, respectively. A simple bucketing algorithm finds local minima of the three breakfasts' summed standard deviations std(S_) + std(H) (light blue, mirrored in the x-axis in the figure) used as bucket borders (vertical grey lines). A lot of the discrepancies disappears in the corresponding bucketed version. The remaining differences in S and $s_{\_i}$ can be used for general and preprandial-postprandial error estimates.

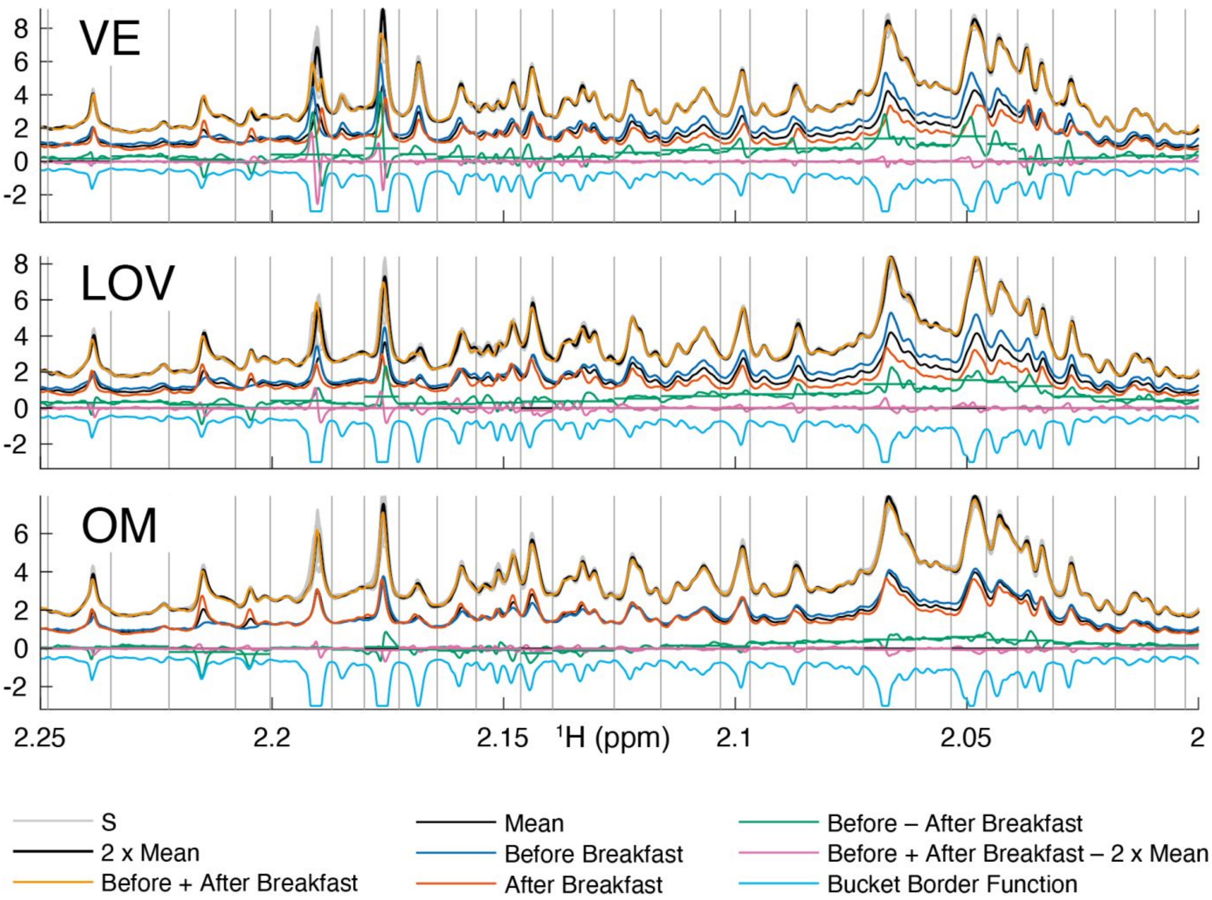

Figure S4. Normalization constant correlation plots. All normalization coefficients were similar independent of method. The most concentrated samples are approximately 9 times stronger than the least. In the final analysis the three persons with the most diluted samples were excluded since they were strong outliers in the PCAs of all three models. The preprandial (blue) vs. postprandial (red) samples had approximately similar concentration distribution making it less likely that these differences bias the final result.

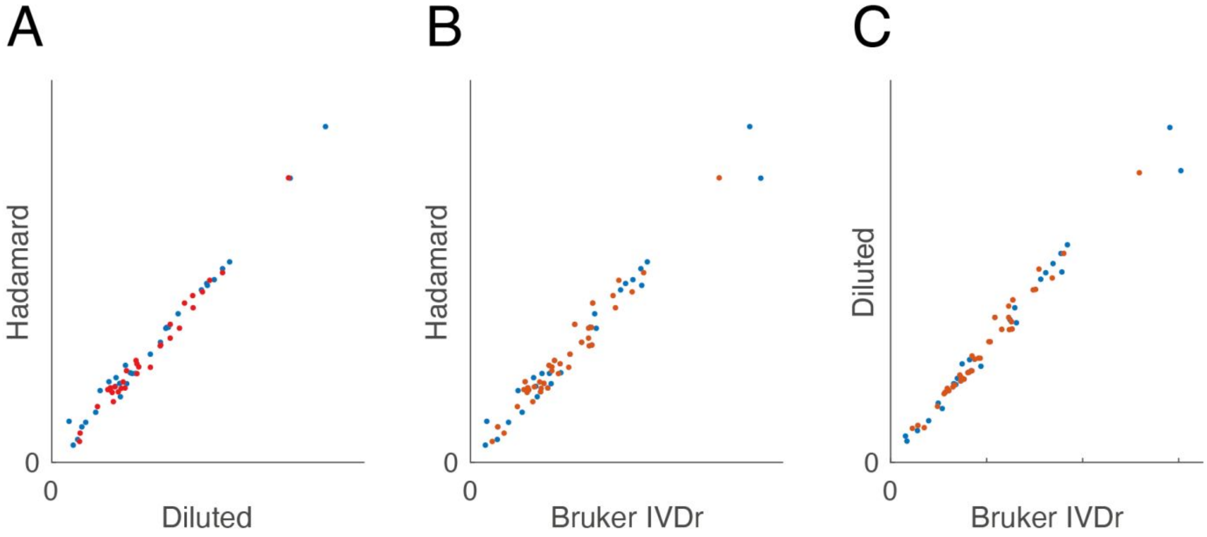

Figure S5. Fumarate. Hadamard calculated (first row) and diluted mean subtracted (second row) bucket intensities for VE (blue) and LOV (red) data of the corresponding bucket at 6.52ppm presented as histograms (left) and experimental sample order (right). The two VE sample blanks do not have any added fumarate while the two LOV blanks at position 47 and 48 have a lot, giving rise to very large peaks compared to all other intensity which is close to zero as shown for the LOV diluted samples (third row, sample blanks in black). All bucket intensities are scaled to the largest of the two LOV sample blank bucket intensities which is set to one (first and second row). For VE, the histograms have similar variability for Hadamard calculated and diluted bucket intensities and a closer inspection show that a large fraction of these intensities is true signal picked up in both Hadamard and diluted spectra and not noise since the Hadamard and diluted VE data is very correlated. The LOV diluted data should be equally good in terms of precision as the VE diluted data and its bucket intensities being close to the true values. The Hadamard calculated data this time deviates from the diluted being much less correlated and with a broader intensity distribution. Especially samples 1, 33, 17 and 49 differ from their corresponding diluted samples. Imperfect pipetting or experimental NMR data acquisition can result in additions and subtractions not fully cancelling each other out in the Hadamard sample scheme. These errors are proportional to the signal inducing it and therefore it is preferable if molecule and sample concentrations are relatively equal in size and not as in this case in the range from very concentrated down to almost noise. If diluting concentrated samples is good in normal schemes to reduce large ppm-shifts, in Hadamard it is also good to even out concentration differences for making the error relative to sample signals similar. Also, it is beneficial to have sample blanks rather than true samples at positions corresponding to experimental time intervals where one thinks it might be difficult to keep the experimental conditions consistent enough and randomize the sample order within each such Hadamard sample block. For example, in this case blanks at positions 1, 33, 17 and 49 seems necessary if one wish to dig out signals close to noise where some other samples have very large signals.

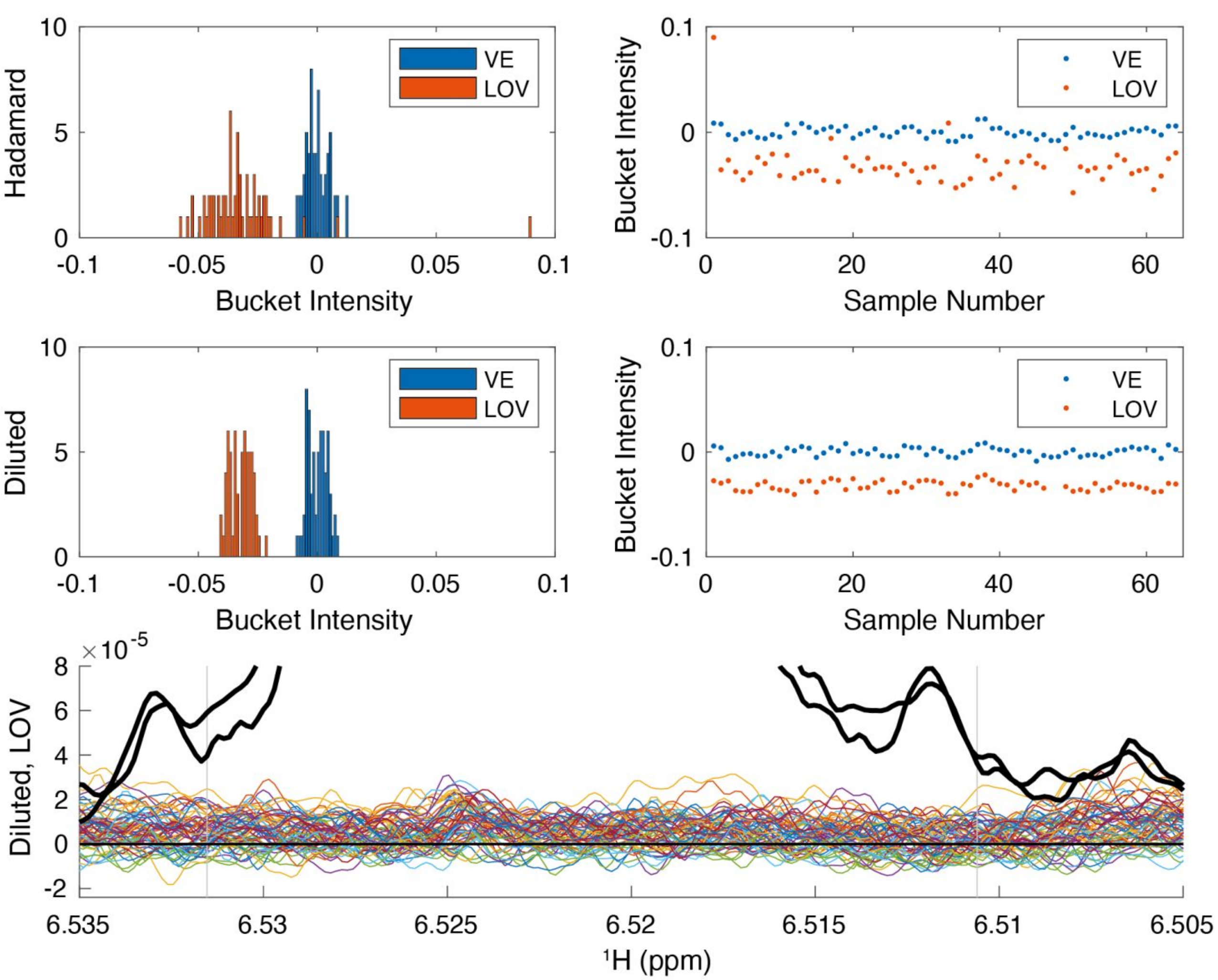
Hadamard
Diluted
Bucket Intensity
Sample Number
VE
LOV
Diluted, LOV
×10^-5
1H (ppm)

Figure S6. OPLS-DA. Loadings with cross validated standard errors from one breakfast's OPLS-EP model vs the loadings with cross validated standard errors from another breakfast's OPLS-EP model using Hadamard, Diluted and Bruker IVDr data. The black color code describes buckets with loading values larger than corresponding cross validated standard errors in at least one of two models but without significant differences between. The blue and red color codes describe the corresponding bucket values from OPLS-DA models of the effect matrix data, blue being significantly higher in one breakfast and red in the other defined as the size of loading values being larger than corresponding cross validated standard errors. The first quadrant has bucket intensities corresponding to metabolites generally increasing after both meals. The second and fourth quadrants show that when some bucket intensities increase after one breakfast they decrease after the other and vice versa. The third quadrant show bucket intensities decreasing after both meals. A few selected buckets are denoted by abbreviated names (see Supplemental Table 1).

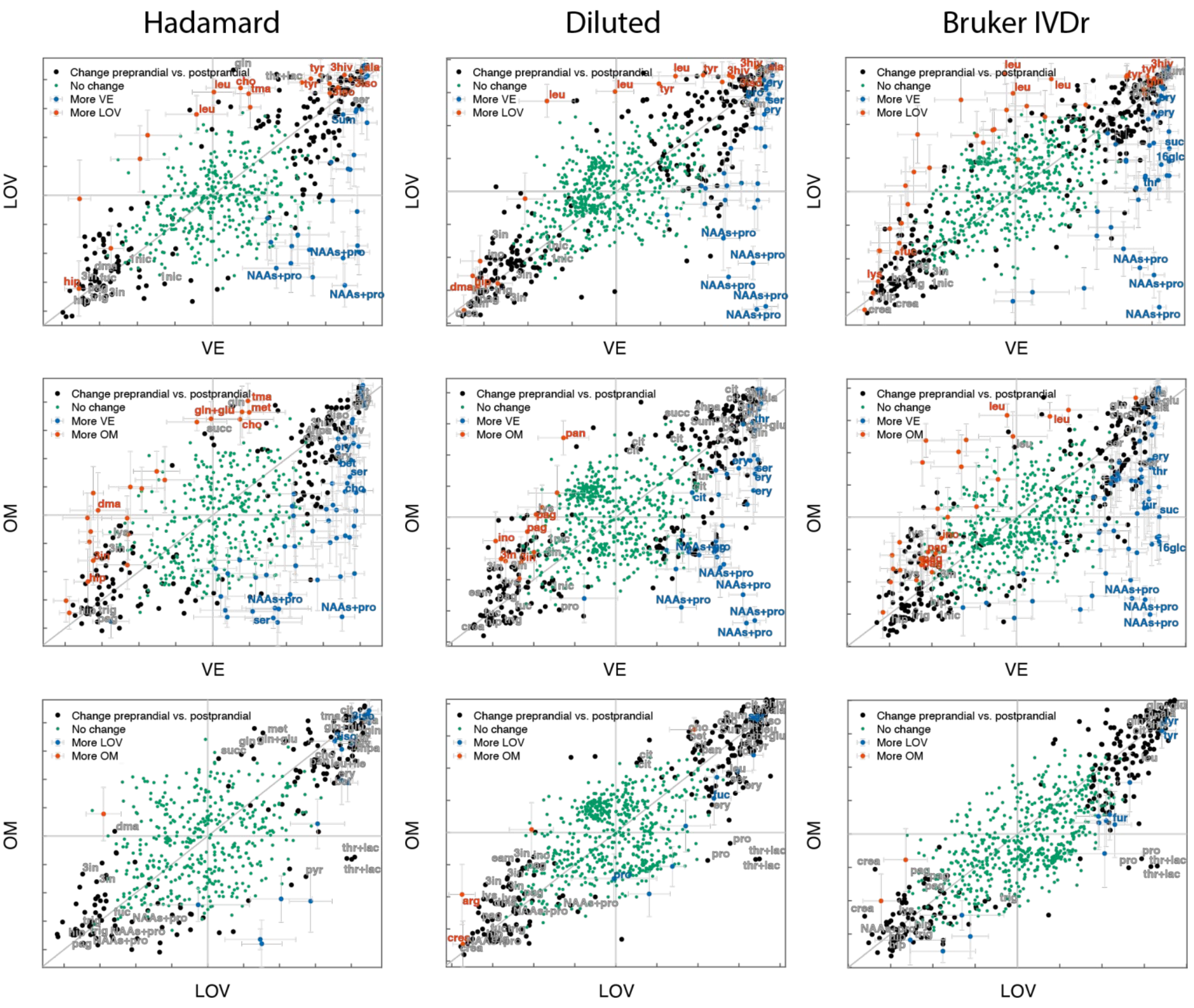

Figure S7. The urea signal intensity is known to be pH dependent around pH 7. The sum $h_{posi} + h_{negi}$ of all pairs should be equal which holds for VE and OM but not LOV meaning that the pH varies over the experiment. Especially the LOV preprandial vs postprandial dimension (yellow) and mean subtracted version (purple) is affected meaning that urea is not consistent when comparing preprandial and postprandial spectra.

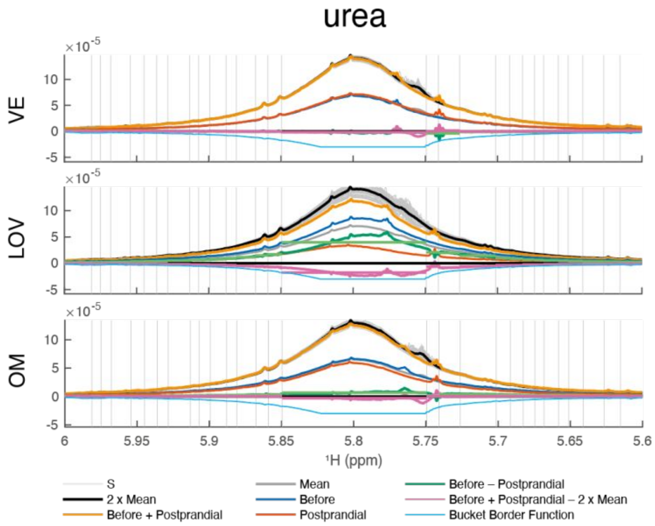

Table S1. Metabolite list

Metabolite	Abbreviation
1-methylnicotinamide	1nic
1,6-anhydro-β-D-glucose	16glc
2-furoate	fur
3-hydroxyisobutyrate or isobutyrate	3iso
3-hydroxyisovalerate	3hiv
3-indoxylsulfate	3in
4-hydroxyphenylacetate	4hpa
alanine	ala
arginine+*unkn*	arg
betaine+*unkn*	bet
choline+*unkn*	cho
citrate	cit
creatinine	crea
dimethylamine	dma
erythritol+*unkn*	ery
ethanolamine	eam
formate	for
fucose+*unkn*	fuc
glutamine	gln
glutamate	glu
glycine	gly
hippurate	hip
inosine	ino
isoleucine	ile
lactate	lac
leucine+*unkn*	leu
lysine	lys
methanol	met
N-phenylacetylglycine	pag
pantothenate	pan
proline	pro
pyruvate	pyr
serine	ser
succinate	succ
sucrose	suc
Sumiki's acid	Sum
threonine	thr
trimethylamine N-oxide	tmao
trigonelline	trig
trimethylamine	tma
tyrosine+*unkn*	tyr
N-acetylated amino acids	NAAs
